# A Potential Probiotic for Diarrhea: *Clostridium tyrobutyricum* Protects Against LPS-Induced Epithelial Dysfunction *via* IL-22 Produced By Th17 Cells in the Ileum

**DOI:** 10.3389/fimmu.2021.758227

**Published:** 2021-11-30

**Authors:** Zhiping Xiao, Lujie Liu, Xun Pei, Wanjing Sun, Yuyue Jin, Shang-Tian Yang, Minqi Wang

**Affiliations:** ^1^ The key Laboratory of Molecular Animal Nutrition, Ministry of Education, College of Animal Sciences, Zhejiang University, Hangzhou, China; ^2^ Department of Chemical and Biomolecular Engineering, The Ohio State University, Columbus, OH, United States

**Keywords:** *Clostridium tyrobutyricum*_1_, diarrhea_2_, intestinal barrier function_3_, IL-22_4_, Th17 cells_5_

## Abstract

Probiotics are clinically used for diarrhea and inflammatory bowel diseases in both humans and animals. Previous studies have shown that *Clostridium tyrobutyricum* (Ct) protects against intestinal dysfunction, while its regulatory function in the gut needs further investigation and the related mechanisms are still not fully elucidated. This study aims to further verify the protective function of Ct and reveal its underlying mechanisms in alleviating diarrhea and intestinal inflammation. Ct inhibited LPS-induced diarrhea and intestinal inflammation in the ileum. IL-22 was identified and the protective role of Ct in the ileum presented an IL-22-dependent manner according to the transcriptomic analysis and *in vivo* interference mice experiments. The flow cytometric analysis of immune cells in the ileum showed that Ct enhanced the proportions of Th17 cells in response to LPS. The results of *in situ* hybridization further verified that Ct triggered Th17 cells to produce IL-22, which combined with IL-22RA1 expressed in the epithelial cells. Moreover, Ct was unable to enhance the levels of short-chain fatty acids (SCFAs) in the ileum, suggesting that the protective role of Ct in the ileum was independent of SCFAs. This study uncovered the role of Ct in alleviating diarrhea and inflammation with the mechanism of stimulating Th17 cells in the lamina propria to produce IL-22, highlighting its potential application as a probiotic for diarrhea and inflammation in the ileum.

## Introduction

Diarrhea is a gastrointestinal condition with high morbidity and mortality. Generally, diarrhea results from the decreased absorption of Cl^-^ and Na^+^ and movement of water, leading to the imbalance of ions and solute across the gut epithelium (1). When diarrhea occurs, the enteric pathogens easily disseminate into the intestinal microenvironment, disturbing homeostasis of the epithelial barrier, host flora, and immune cells, inducing intestinal inflammation, causing intestinal inflammation, and gastrointestinal diseases like inflammatory bowel disease (IBD).

Alleviation of diarrhea and intestinal inflammation requires an ingenious intestinal barrier system concerting the crosstalk between the microbiota and immune cells to avoid excessive immune responses to commensal microbes or pathogens. This process involves a series of mechanisms, including the secretion of cytokines, chemokines, and immunomodulatory molecules, such as thymic stromal lymphopoietin, TGF-β, retinoic acid, IL-10, and IL-5 produced by intestinal epithelial cells (IECs) (2). The mechanisms involved in microbiota-IECs-immune cell interactions are still being elucidated because of the cell culture systems imitating the physiological cues of the complex gastrointestinal mucosa microenvironment. However, equilibrium of the gut microbiota, IECs, and immune cells is crucial for intestinal homeostasis.

*Clostridium tyrobutyricum* (*C. tyrobutyricum*/Ct) is one of the anaerobic *Clostridium* strains. Compared with other gram-positive bacteria, the cell well of *C. tyrobutyricum* is mainly made up of higher protein (35-40% dry weight), lower insoluble peptidoglycan (10-12% dry weight), and neutral sugar, and absent teichuronic acids (3, 4). Numerous studies have revealed that *Clostridium tyrobutyricum* could be used to produce higher butyric acid *via* bacterial fermentation (5–7). Recently, *C. tyrobutyricum* has been revealed to protect barrier function in different tissues, including the gut (8), testis (9), and endometrium (10). A new study about the genomic analysis of *C. tyrobutyricum* pointed out that *C. tyrobutyricum* is a potential bacteria in regulating health in human beings (11). Our earlier study has shown that *C. tyrobutyricum* protects against lipopolysaccharide (LPS)-induced epithelial dysfunction in IPEC-J2 cells (12). Moreover, *C. tyrobutyricum* has been revealed in alleviating LPS-induced inflammation *via* regulating immune cells in the intestinal sections (13, 14). However, the regulatory function of *C. tyrobutyricum* in the gut needs further elucidation and its underlying mechanism in maintaining intestinal homeostasis is still not fully understood.

In this study, we aimed to investigate the role and possible mechanisms of *C. tyrobutyricum* in the ileum based on our previous study (14). We further proved that *C. tyrobutyricum* prevented diarrhea and protected against LPS-induced ileal barrier dysfunction *in vivo*. IL-22 was identified according to the transcriptomic analysis of the ileum. *C. tyrobutyricum* alleviated LPS-induced epithelial destruction depending on IL-22 produced according to the *in vivo* interference experiments. We also revealed that *C. tyrobutyricum* enhanced the MHC-II process, thereby stimulating Th17 cells to produce IL-22 which combined with IL-22RA1 expressed in the epithelial cells. Strikingly, *C. tyrobutyricum* decreased the levels of short-chain fatty acids (SCFAs) in response to LPS, suggesting that the regulatory function of *C. tyrobutyricum* in the gut was independent of SCFAs. Our studies revealed the role of *C. tyrobutyricum* in the crosstalk between epithelial cells and immune cells mediated by IL-22 and identifies the potential application of *C. tyrobutyricum* as a probiotic for diarrhea and inflammation in the ileum.

## Materials and Methods

### Antibodies and Reagents

LPS (derived from Escherichia coli strain O55:B5) and Collagenase VIII from *Clostridium* histolyticum (C2139) were from Sigma-Aldrich (USA). The antibodies in the flow cytometry were described as our previous studies (13, 14). All antibodies used in the flow cytometric analysis, immunoblotting, and immunohistochemistry were shown in **Supplemental Table 1**. Transcription Factor Staining Buffer Set (562574) and Stain Buffer FBS (554656) were from BD (USA). HEPES was from Gibco (USA). EDTA and *Bac*Light™ Green Bacterial Stain (B-35000) were from Invitrogen (USA). Fetal Bovine Serum was purchased from Gemini (USA). RMPI 1640 was from BI (Israel). TRIzol reagent, The PrimeScript RT reagent kit with gDNA Erase, and SYBR Green qPCR reagent were from Takara (Japan). DNase (D8070), 5% BSA (SW3015), Triton X-100 (P1080) and DAPI (C0065) were from Solarbio (China). The Opal 7-color Manual IHC kit (NEL811001KT) was purchased from PerkinElmer (USA). Polybrene was provided by HanBio (China). The whole cell lysis assay (KGP2100) was from KeyGEN (China).

### Mice

Four-week-old C57BL/6 male mice were purchased from Shanghai SLAC Laboratory Animal Co., Ltd and bred at the Zhejiang University Laboratory Animal Center (25°C, 12/12-h light/dark cycle) allowing unrestricted access to standard mice diet and water. The mice experiments were performed following the protocol approved by the Institutional Animal Care and Use Committee of Zhejiang University. For the mice feeding trial, four-week C57BL/6 male mice were treated with PBS (Control, mice were treated with PBS by gavage for 20 days followed by intraperitoneal injection with PBS, n=10), Ct (mice were treated with 10^8^ CFU/mL Ct for 20 days followed by intraperitoneal injection with PBS, n=10), LPS (mice were treated with PBS for 20 days followed by intraperitoneal injection with 10 mg/kg BW LPS, n=12), and Ct + LPS (mice were treated with 10^8^ CFU/mL Ct for 20 days followed by intraperitoneal injection with 10 mg/kg BW LPS, n=12). For the *in vivo* interference feeding experiment, adeno-associated virus 2/2-m-shIL22 labeled with GFP (AAV-shIL22) and AAV-shNC were generated by Hanbio (Shanghai, China). The sequences of AAV-shNC and AAV-shIL22 were designed as the previous study, namely 5’-TTCTCCGAACGTGTCACGTAA-3’ and 5’-GCTAAGGATCAGTGCTACCTGATGA-3’ (13). Four-week C56BL/6 male mice were first intraperitoneally injected with 200 μL AAV-shIL22/AAV-shILNC at a concentration of at least 2.4 × 10^11^ vg/mL. After 14 days, mice were treated with PBS (AAV-shNC-Control, n=19; AAV-shIL22-Control, n=17), Ct (AAV-shNC-Ct, n=14, AAV-shIL22-Ct, n=12), LPS (AAV-shNC-LPS, n=20; AAV-shIL22-LPS, n=18), and Ct + LPS (AAV-shNC-Ct + LPS, n=17; AAV-shIL22-Ct + LPS, n=12), respectively as described above.

### Bacterial Culturing

*C. tyrobutyricum* was provided by Prof. Shang-Tian Yang, from The Ohio State University and was cultured as our previous study (5). Simply, *C. tyrobutyricum* was cultured anaerobically at 37°C in a clostridial growth medium (CGM), collected after centrifugation at 12,000 rpm for 5 min, and suspended in PBS.

### Bacterial Colonization

For the colonization of *C. tyrobutyricum* in the intestine, 10^8^ CFU/mL *C. tyrobutyricum* was first mixed with 100 μM working solution of the *Bac*Light bacterial stain, incubated for 15 min at room temperature, and washed with PBS. C57BL/6 mice were then treated with *C. tyrobutyricum* by gavage and the intestinal samples were collected, frozen with liquid nitrogen, embedded with OCT, and sectioned. The fluorescence was visualized using a confocal microscope LSM 880 and captured using ZEN 2.3 software (Carl Zeiss, Germany).

### RNA-Sequencing

Total RNA of ileal samples from the Control, LPS, Ct, and Ct + LPS groups (n=6) were isolated for RNA-Sequencing. RNA sequencing was performed by Novogene using an Illumina sequencer. Clean reads obtained by removing reads containing adapter, poly-N and low-quality reads were aligned to the reference genome using Tophat2 RNA-Seq alignment software. HTSeq was used to count the read numbers mapped to each gene. Differential expression analysis was conducted using the DESeq R package. The P values were adjusted using the Benjamini & Hochberg method (FDR). Corrected P-value of 0.05 and fold-change of at 1 was set as the threshold for significantly differential expression.

### Immune Cells Isolation and Flow Cytometric Analysis

The isolation of immune cells from the ileal lamina propria was performed as previously described (15, 16). The steps of cell isolation and antibodies staining were conducted according to our previous studies (13, 14). Simply, the ileal sections were isolated from the mice, washed with PBS, cut into 5-cm pieces after removing the fat tissue, mesenteric, and Peyer’s patches, and shaken in PBS containing 2% FBS, 10 mM HEPES, and 2 mM EDTA at 37°C for 30 min for two to three cycles. The intestinal pieces were then shaken in RPMI 1640 medium containing 10% FBS, 2 mM L-glutamine, 100 U/mL penicillin, 100 μg/mL streptomycin, 0.6 mg/mL collagenase VIII, and 150 μg/mL DNase at 37°C for 30 min for two cycles, passed through 100 μm and 40 μm cell strainers, and centrifuged at 400 × g for 10 min. Cells were then stained with Live/Dead (FVS780) for 20 min followed by CD16/32 antibody for 15 min, washed with PBS, stained with surface antibodies for 20 min, and washed with PBS. For the intracellular antibodies, cells were fixed and permeabilized for 2 h followed by staining the intracellular antibodies for 2 h, washed with PBS, and suspended in PBS before processing. The data were analyzed with FlowJo software (BD, USA).

### Transmission Electron Microscopy and Scanning Electron Microscopy

Ileal sections were washed in PBS and fixed in 2.5% GA overnight. All samples were washed in PBS and postfixed in 1% osmic acid for 1-2 h. Then the samples were washed in PBS and dehydrated in a series of gradient ethanol solutions (50%, 75%, 85%, 95%, and 100% ethanol), each for 15 min. For SEM, the samples were dehydrated in a Hitachi Model HCP-2 critical point dryer with liquid CO_2_ and visualized using a Philips Model SU8010 FASEM (Hitachi, Japan). For TEM, samples were embedded in Epon resin and cut into 60 nm ultrathin sections. Sections were counterstained with uranyl acetate and lead citrate. All the samples were observed using a Hitachi HT7650 electron microscope (Hitachi, Japan).

### Histology and Immunohistochemistry

Ileal samples were fixed overnight in 4% PFA, and then dehydrated in 30% sucrose in PBS solution for 48 h until sunk to the bottom. Ileal sectioning, H&E, and ZO-1 staining were performed by Zhejiang Chinese Medical University. The scanning was conducted on Nikon Eclipse 80i (Nikon, Japan). The intestinal morphology was evaluated with NDP. View2 (Hamamatsu, China). The average of density (AOD) of ZO-1 was obtained with ImageJ software.

### *In situ* Hybridization

For tissue fluorescence *in situ* hybridization, the Opal 7-color Manual IHC kit was used according to the manual provided. The sections were dewaxed with xylene, rehydrated through a graded series of ethanol solutions, and fixed in 10% neutral buffered formalin. After microwave treatment and blocking, the ileal sections were sequentially stained with primary antibodies and HRP-conjugated secondary antibodies. One of four Opal reagents was used for staining followed by microwave treatments and another round of staining. Slides were finally stained with DAPI for 5 min and mounted before image acquisition. CD45, EpCAM, CD3e, RORγt, IL-22, and IL-22RA1 were used as primary antibodies. The dyes Opal 520, Opal540, Opal570, and Opal 590 were used for staining. Images were visualized using confocal microscope LSM 880 and captured using ZEN 2.3 software (Carl Zeiss, Germany). Considering the autofluorescence of tissues, the contrast/brightness with consistent parameters was regulated in all images using ZEN 2.3 software. All images were analyzed with ImageJ software.

### RT-qPCR

Total RNA was extracted using TRIzol reagent. cDNA was synthesized with PrimeScript RT reagent kit with gDNA Eraser according to the manufacturer’s instructions. RT-qPCR was performed on the CFX96TM Real-Time System (Bio-Rad) in duplicate or triplicate. Data were analyzed according to the 2^-△△Ct^ method and normalized to the expression of GAPDH. PCR primers are shown in **Supplemental Table 2**.

### Immunoblotting

Whole-cell lysates of ileum was prepared using Whole Cell Lysis Assay. After electrophoresis with 10% SDS-PAGE, proteins were transferred to PVDF membranes. The membranes were blocked with 5% non-fat milk for 1h at room temperature, incubated with specific primary antibodies followed by HRP-conjugated secondary antibodies, and detected by the ECL reagent.

### Gas Chromatographic Analysis

The concentrations of SCFAs were analysed *via* gas chromatography (17, 18) The ileal contents were mixed with 5 mL double distilled water for 1 h. The supernatant was collected after centrifugation at 10,000 g for 15 min and mixed with 85% orthophosphoric acid for 1 h. The supernatant was then collected, passed by 0.22 μm strainers, and transferred into the gas chromatography vial. The concentrations of SCFAs in the ileal contents of mice were measured by GC-8A gas chromatography (Shimadzu, Kyoto, Japan).

### Statistical Analysis

All statistical tests were performed with Prism 8.0 software and analyzed using two-tail unpaired t-test. All data are expressed as mean ± SEM and *P* < 0.05 was considered significant and the level of significance was indicated as *P < 0.05, **P < 0.01.

## Results

### Ct Alleviates LPS-Induced Ileal Barrier Dysfunction

Our previous studies have investigated the most efficient concentration of Ct in alleviating intestinal dysfunction and suggested that 10^8^ CFU/mL Ct effectively alleviated LPS-induced inflammation in different sections of the intestine including the duodenum, ileum, and colon (13, 14). To further understand the role of Ct in alleviating diarrhea and intestinal inflammation *in vivo*, we first evaluated the colonization of Ct *in vivo via* staining Ct with *Bac*Light™ green. Mice were treated with 10^8^ CFU/mL Ct by gavage. After 4 h, faint fluorescence was observed on the lumen and the villus surface of the small intestines (**Supplemental Figure 1**), indicating that Ct could colonize in the small intestines, while its colonization capacity in the intestine was weak.

**Figure 1:**
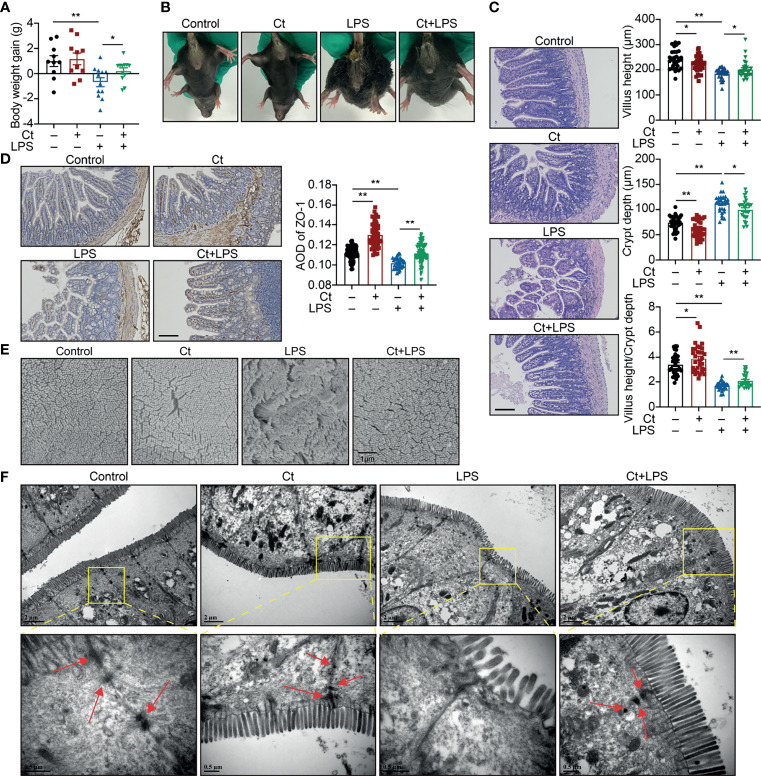
Ct alleviates LPS-induced diarrhea and ileal barrier function *in vivo*. C57BL/6 were randomly divided into 4 groups, including the Control (n=10), Ct (n=10), LPS (n=12), Ct + LPS (n=12). All samples were collected after 24 h. **(A)** Body weight gain. **(B)** Diarrhea. **(C, D)** H&E staining **(C)** and ZO-1 expression **(D)**. The presented figures of H&E and ZO-1 staining in each group were from the same sample. Scale bar: 500 μm. **(E)** Visualization of villus morphology under SEM. Scale bar: 1 μm. **(F)** Visualization of microvillus and structure of tight junctions under TEM. Scale bar: 2 μm. Data were presented as mean ± SEM. The significant difference was analyzed by two-way unpaired t-tests. *P < 0.05, **P < 0.01.

Mice were then treated with 10^8^ CFU/mL Ct by gavage followed by LPS injection. Compared with LPS group, Ct maintained the body weight gain (**Figure 1A**) and inhibited diarrhea (**Figure 1B**) in response to LPS. In response to LPS, Ct improved the ileal morphology, enhanced the villus height, and villus height/crypt depth ratio (**Figure 1C**), which were consistent with our previous study (14). Compared with LPS group, Ct significantly increased the expression of ZO-1 (a typical tight junction protein) in the ileum in response to LPS (**Figure 1D**). Under electron microscopy, we observed that LPS induced the destruction of villi, microvilli, and tight junction structures, while Ct improved these phenomena in response to LPS (**Figures 1E, F**). These results further verified that Ct effectively inhibited diarrhea and alleviated ileal barrier dysfunction induced by LPS.

### Ct Enhances the Expression of IL-22 in the Ileum

**Figure 2:**
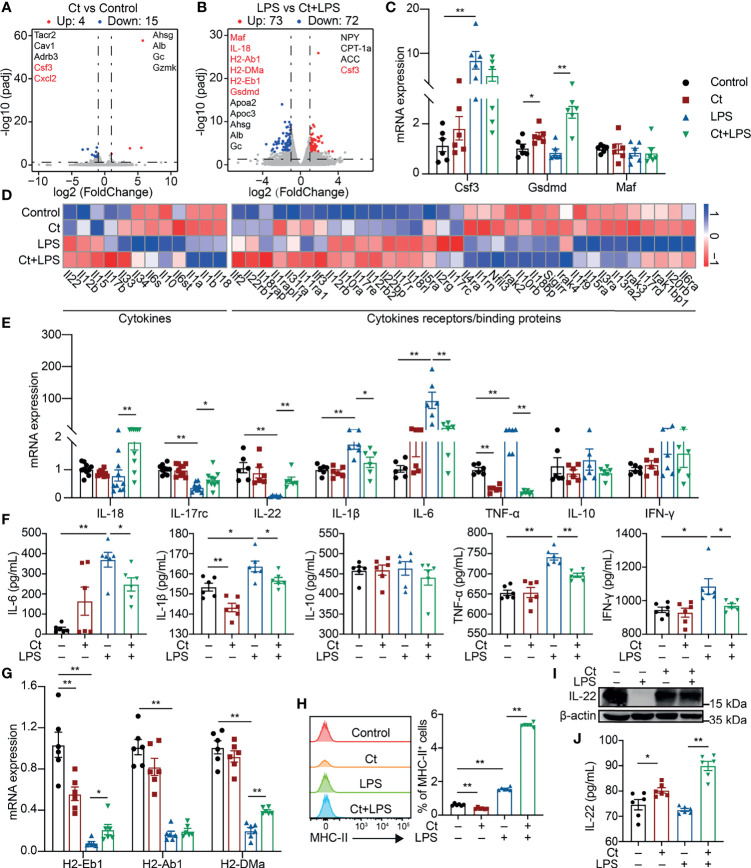
Ct enhances the expression of IL-22 in response to LPS. **(A)** Volcano plots of differentially expressed genes between the Control and Ct group (Ct *vs* Control, n=6). **(B)** Volcano plots of differentially expressed genes between the LPS and Ct + LPS group (LPS *vs* Ct + LPS, n=6). **(C)** RT-qPCR analysis of Csf3, Gsdmd, and Maf in the ileum (n=6). **(D)** Heat-map of differentially expressed genes enriched in cytokines and cytokine receptors/binding proteins. **(E)** RT-qPCR analysis of cytokines including IL-18, IL-17rc, IL-22, IL-1β, IL-6, TNF-α, IL-10, and IFN-γ in the ileum (IL-18 and IL-17rc, n=10, others, n=6). **(F)** The levels of inflammatory cytokines including IL-6, IL-1β, IL-10, TNF-α, and IFN-γ in the ileum (n=6). **(G)** RT-qPCR analysis of MHC-II transcripts in the ileum (n=6). **(H)** Proportions of MHC-II^+^ cell in the ileum (n=6). **(I)** Immunoblotting analysis of IL-22 in the ileum. **(J)** ELISA analysis of IL-22 levels in the ileum (n=6). Data were presented as mean ± SEM. The significant difference was analyzed by two-way unpaired t-tests. *P < 0.05, **P < 0.01.

The ileal samples were collected after the feeding experiment and transcriptomic analysis was conducted to elucidate the molecular mechanism of Ct in the ileum. A total of 19 differentially expressed genes were identified between the Ct and Control groups according to padj < 0.05 and |log_2_(FoldChange)| > 1 (**Figure 2A** and **Supplemental Table 3**). Ct up-regulated genes like Ahsg and Alb, which have been revealed in inhibiting intestinal diseases such as ulcerative colitis (19, 20). In addition, Ct down-regulated genes including Tacr2, Cav1, Adrb3, Csf3, and Cxcl2, which enriched in ‘Neuroactive ligand-receptor interaction’, ‘Calcium signaling pathway’, ‘Endocytosis’, and ‘Cytokine-cytokine receptor’ processes (**Supplemental Figure 2A** and **Supplemental Table 4**). These results indicated that Ct inhibited intestinal inflammation and regulated intestinal nervous response. A total of 145 differentially expressed genes were identified between the LPS and Ct + LPS groups (**Figure 2B**, **Supplemental Table 5**). Compared with the LPS group, genes including Maf, IL-18, H2-Ab1, H2-DMa, H2-Eb1, Gsdmd, Ahsg, Alb, and Gc enriching in ‘Intestinal bowel disease’ and bacterial infection were enhanced in the Ct + LPS group. In addition, Ct regulated the intestinal metabolism, especially fatty acid metabolism in response to LPS (**Supplemental Figure 2B** and **Supplemental Table 6**). Altogether, we suggested three possible mechanisms of Ct in regulating intestinal immune response, namely neuro-, metabolism-, and cytokines-immune regulation. In this study, we focused on the mechanism of cytokines-immune regulation.

The mRNA expression of Csf3, Gsdmd, and Maf was first determined. No differences in the mRNA expression of Csf3, Gsdmd, and Maf were observed between the LPS and Ct + LPS groups (**Figure 2C**), which were inconsistent with the transcriptomic analysis. In the innate immune system, pathogens/extracellular stimuli are recognized by pattern-recognition receptors (PRRs) like Toll-like receptors (TLRs) and NOD-like receptors (NLRs) to induce the recruitment of monocytes, thereby producing cytokines and chemokines (21, 22). According to the transcriptomic analysis, we also noticed that Ct decreased the expression of TLRs such as TLR4, a well-known receptor of LPS (**Supplemental Figure 3A, Supplemental Table 7**). *Clostridium butyricum*, another *Clostridium* strain, which has been clinically used in regulating intestinal health with the mechanism *via* activating TLR2/MyD88 signaling pathway in colitis (23). Unlike *Clostridium butyricum*, Ct decreased the expression of TLR2 in response to LPS, indicating that Ct regulated intestinal health in a TLR2-independent manner. NLRP9b is highly expressed in the ileal epithelial cells and restricts rotavirus infection (24). In this study, Ct decreased the mRNA expression of NLRP6 and NLRP9b in response to LPS (**Supplemental Figure 3B**).

To address whether Ct regulated intestinal immune response *via* cytokines. Herein, some differentially expressed cytokines and chemokines were identified among the Control, Ct, LPS, and Ct + LPS groups (**Figure 2D** and **Supplemental Tables 8, 9**). We then analyzed the mRNA expression of these cytokines and chemokines in the ileum using RT-qPCR. Ct decreased the mRNA expression of IL-1β, IL-6, TNF-α, Ccl2, Ccl9, and Ccl11 in response to LPS (**Figure 2E** and **Supplemental Figure 3D**). Besides, in response to LPS, Ct decreased the levels of IL-6, IL-1β, TNF-α, and IFN-γ in the ileum according to ELISA analysis (**Figure 2F**). The above results showed that Ct inhibited LPS-induced inflammation in the ileum. We also noticed that Ct enhanced the mRNA expression of IL-18, IL-17rc, and IL-22 in response to LPS (**Figure 2E**). Some earlier studies have shown that these three cytokines (IL-18, IL-17, and IL-22) induce intestinal inflammation (25–27), while recent studies show the protective roles of these cytokines in the gut (28–30). These guided us to hypothesize that Ct might regulate intestinal inflammation *via* enhancing IL-18, IL-17, and IL-22 in the ileum.

According to the results of RNA-seq and RT-qPCR verification, in response to LPS, Ct enhanced the mRNA expression of H2-Eb1 and H2-DMa in the ileum (**Figure 2G**), which have been defined as major histocompatibility complex class II-related transcripts (MHC-II) (31). Generally, MHC-II is constitutively expressed on antigen-presenting cells and epithelial cells, recognizing the antigen derived from nutrients, commensal bacteria, or pathogens, and is sensitized by CD4^+^ T cells, thereby promoting immune response (32–35). MHC-II deficiency accounts for higher susceptibility to enteric infections, leading to intestinal inflammation and gastrointestinal disease like IBD (36). We then analyzed the proportions of MHC-II^+^ cells in the ileum. Compared with LPS group, Ct significantly enhanced the proportion of MHC-II^+^ cells in response to LPS (**Figure 2H**). It has been reported that MHC-II, IL-22, and type 3 innate lymphoid cells (ILC3s) partially overlap in the intestine according to the sing-cell RNA-sequencing (37), which guided us to assume that Ct might regulate intestinal immune response *via* IL-22. We then measured the protein expression of IL-22 in the ileum and found that Ct enhanced the IL-22 expression in response to LPS (**Figure 2I**). The level of IL-22 in the ileum was also measured using ELISA. In response to LPS, Ct enhanced the level of IL-22 in the ileum (**Figure 2J**). Altogether, Ct might alleviate LPS-induced inflammation *via* IL-22 in the ileum.

### Ct Protects LPS-Induced Inflammation Depending on IL-22 in the Ileum

To investigate whether Ct protected intestinal barrier function and alleviated LPS-induced intestinal inflammation depending on IL-22. An RNA interference experiment *in vivo* with adeno-associated virus-shIL22 labeled with GFP (AAV-shIL22) by intraperitoneal injection was conducted to knock down IL-22 in the intestine. The fluorescence was observed on the surface of both ileum and colon, offering the possibility for AAV-shIL22 to target the intestine (**Supplemental Figure 4A**). The mRNA expression of IL-22 in the ileum was reduced in mice treated with AAV-shIL22 and the efficiency of *in vivo* interference reached 64.19% (**Supplemental Figure 4B**).

**Figure 3:**
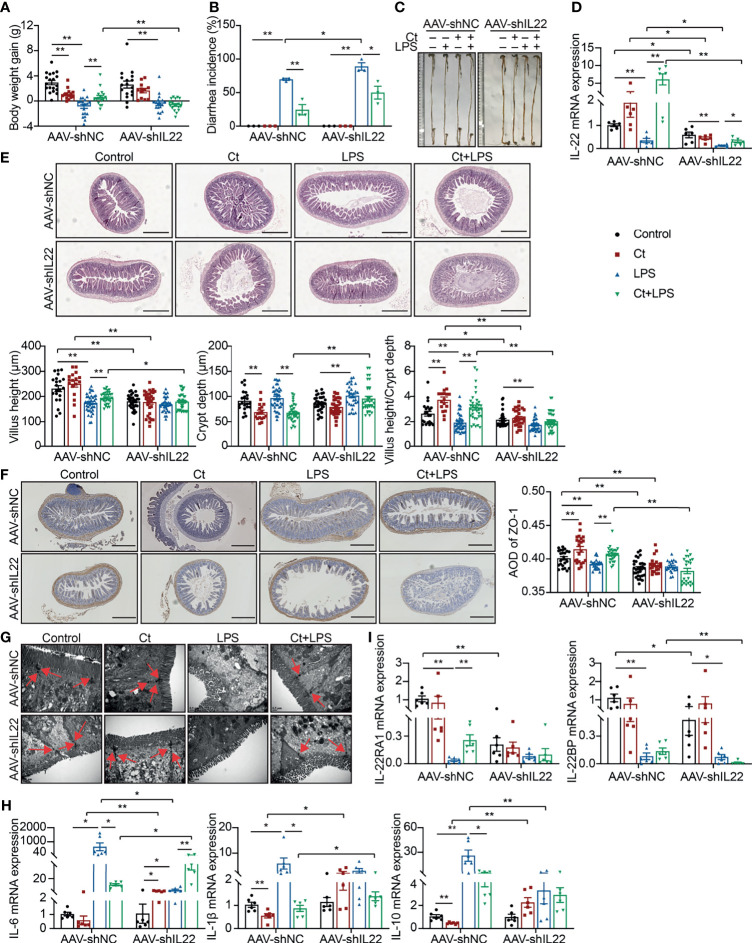
Ct alleviates intestinal barrier dysfunction in an IL-22-dependent manner. C57BL/6 mice were first intraperitoneally injected with AAV-shNC (n=70) and AAV-shIL22 (n=59), after 14 days, mice were randomly divided into 8 groups, namely AAV-shNC-Control (n=19), AAV-shNC-Ct (n=14), AAV-shNC-LPS (n=20), AAV-shNC-Ct + LPS (n=17), AAV-shIL22-Control (n=17), AAV-shIL22-Ct (n=12), AAV-shIL22-LPS (n=18), AAV-shIL22-Ct + LPS (n=12). All samples were collected after 24 h. **(A)** Body weight gain. **(B)** Diarrhea incidence. **(C)** Intestinal length. **(D)** RT-qPCR analysis of IL-22 in the ileum (n=6). **(E)** H&E staining. Scale bar: 500 μm. **(F)** ZO-1 expression. Scale bar: 500 μm. **(G)** Visualization of microvilli and structure of tight junctions under TEM. Scale bar: 0.5 μm. **(H)** RT-qPCR analysis of inflammatory cytokines including IL-6, IL-1β, and IL-10 in the ileum (n=6). **(I)** RT-qPCR analysis of IL-22 and IL-22BP in the ileum (n=6). Data were presented as mean ± SEM. The significant difference was analyzed by two-way unpaired t-tests. *P < 0.05, **P < 0.01.

After knocking down IL-22, Ct was unable to maintain the body weight gain (**Figure 3A**), while Ct decreased the diarrhea incidence in response to LPS (**Figure 3B**). In mice treated with AAV-shNC, Ct maintained the whole length of the intestine in response to LPS, while no differences were observed between LPS and Ct + LPS groups after knocking down IL-22 (**Figure 3C**). We also observed the above phenomenon in the colon and demonstrated that Ct alleviated colonic dysfunction in an IL-22-dependent manner (13). After knocking down IL-22, compared with LPS group, Ct still enhanced the mRNA expression of IL-22 in the ileum in response to LPS (**Figure 3D**). Fragmentary villi were observed after knocking down IL-22 in the ileum, and in response to LPS, Ct dramatically decreased the villus height and villus height/crypt depth ratio after knocking down IL-22 compared with that in AAV-shNC-Ct + LPS group. After knocking down IL-22, Compared with LPS group, Ct was unable to enhance the villus height and villus height/crypt depth ratio in response to LPS (**Figure 3E**).

Compared with mice treated with AAV-shNC, the expression of ZO-1 in the Control, Ct, and Ct + LPS groups was decreased after knocking down IL-22, and Ct was unable to maintain ZO-1 expression in mice treated AAV-shIL22 in response to LPS (**Figure 3F**). Although the structure of TJs was observed, the microvilli was damaged in the AAV-shIL22-Ct + LPS group (**Figure 3G**). Besides, after knocking down IL-22, Ct enhanced the mRNA expression of IL-6 and had no effects on IL-1β and IL-10 in response to LPS (**Figure 3H**). Collectively, these findings indicated that Ct protected against LPS-induced dysfunction depending on IL-22 in the ileum.

The IL-22 receptor (IL-22R), composed of two heterodimeric subunits including IL-22RA1 and IL-10R2, is one of the IL-10 family of receptors (38). According to binding studies, IL-22 initially binds to IL-22RA1 since IL-22 has a high affinity for IL-22RA1, and as such, the formation of the IL-22-IL-22RA1 complex enables secondary binding of the IL-10R2 subunit to activate downstream signaling (39). In this study, after knocking down IL-22, although Ct enhanced the mRNA expression of IL-22 in response to LPS, no differences in the mRNA expression of IL-22RA1 in the ileum were observed between the LPS and Ct + LPS groups. IL-22BP, a soluble form of the IL-22RA1 subunit with a higher affinity to IL-22 than the membrane-bound IL-22RA1 form, binds to IL-22 at an overlapping site to IL-22RA1 (40, 41). Compared with LPS group, Ct had no effects on mRNA expression of IL-22BP in response to LPS (**Figure 3I**).

### Ct Stimulates Th17 Cells to Produce IL-22 in the Lamina Propria

IL-22, identified as one of the IL-10 members, is generally produced by a series of immune cells such as Th17 cells, ILC cells, and dendritic cells (DCs) (42, 43). According to the transcriptomic analyses, we identified some differentially expressed genes enriched in the cluster of differentiation (**Supplemental Figure 3E**), some of which have been defined as the surface biomarkers of immune cells, indicating that Ct might specifically regulate the function of intestinal immune cells. Our previous study also points out that Ct mainly targets Th17 cells in the ileum and supposes that Ct may stimulate Th17 cells to produce IL-22 in the ileum (14).

**Figure 4:**
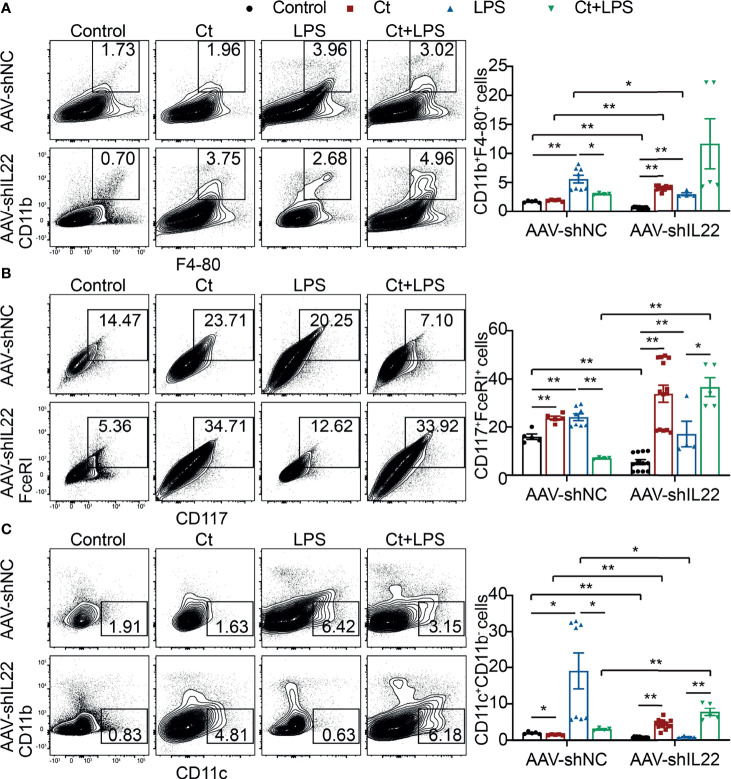
Effects of Ct on the proportions of macrophages, mast cells, and DCs in the ileum. Flow cytometric analyses were conducted on 8-colour FACS Verse and three panels were used to analyzing the intestinal immune cells. The single cells were first gated on SSC-A *vs* FSC-A and FSC-A *vs* FSC-H, and then CD45^+^FVS780^-^ cells (CD45^+^Live^+^ cells) were gated out. CD11b^+^F4-80^+^ cells, CD117^+^FceRI^+^ cells, and CD11c^+^CD11b^-^ cells were gated out from CD45^+^Live^+^ cells. **(A)** Proportions of macrophages (CD11b^+^F4-80^+^ cells). **(B)** Proportions of mast cells (CD117^+^FceRI^+^ cells). **(C)** Proportions of DCs (CD11c^+^CD11b^-^ cells). Each point presented a mouse. Data were presented as mean ± SEM. The significant difference was analyzed by two-way unpaired t-tests. *P < 0.05, **P < 0.01.

**Figure 5:**
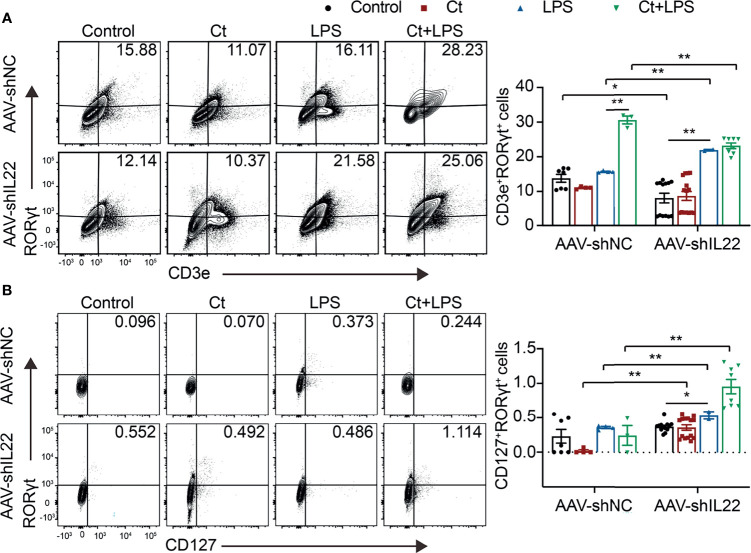
Ct stimulates Th17 cells rather than ILC3s in the ileum. Flow cytometric analyses were conducted on 8-colour FACS Verse and three panels were used to analyzing the intestinal immune cells. The single cells were first gated on SSC-A *vs* FSC-A and FSC-A *vs* FSC-H, and then CD45^+^FVS780^-^ cells (CD45^+^Live^+^ cells) were gated out. CD3e^+^RORγt^+^ cells were gated out from CD45^+^CD3e^-^ cells. CD45^+^CD3e^-^ cells were gated out from CD45^+^FVS780^-^ cells and CD127^+^RORγt^+^ cells were gated out from CD45^+^CD3e^-^ cells. **(A)** Proportions of Th17 cells (CD45^+^CD3e^+^RORγt^+^ cells). **(B)** Proportions of ILC3s (CD45^+^CD3e^-^CD127^+^RORγt^+^). Each point presented a mouse. Data were presented as mean ± SEM. The significant difference was analyzed by two-way unpaired t-tests. *P < 0.05, **P < 0.01.

To investigate whether Ct regulates ileal immune cells in the *in vivo* interference feeding experiment, we isolated ileal lamina propria cells from AAV-shNC- and AAV-shIL22-treated mice to analyze the proportions of immune cells including macrophages, mast cells, DCs, ILC3s, CD4^+^ T cells, and T cell subsets in the ileum. As shown in **Figure 4** and **Supplemental Table 10**, in the innate immune system, In mice treated with AAV-shNC, compared with LPS group, Ct decreased the proportions of macrophages, mast cells, and DCs in response to LPS in the ileum, which were consistent with our previous study (14). After knocking down IL-22, Ct enhanced the frequencies of mast cells and DCs in response to LPS. Numerous studies have revealed that IL-22 is induced by immune cells such as Th17 cells and ILC3s, thereby repairing epithelial damage (38, 44–48). Herein, as shown in **Figure 5A**, **Supplemental Tables 11, 12** and **Supplemental Figure 5**, in mice treated with AAV-shNC, in response to LPS, Ct increased the proportions of Th1 cells, Tregs, and Th17 cells, while after knocking down IL-22, Ct decreased the numbers of Th1 cells and Tregs and had no effects on Th17 cells number. Besides, compared with LPS, Ct had no effects on the proportions of ILC3s in response to LPS (**Figure 5B**), indicating that Ct triggered Th17 cells rather than ILC3s in the ileum.

**Figure 6:**
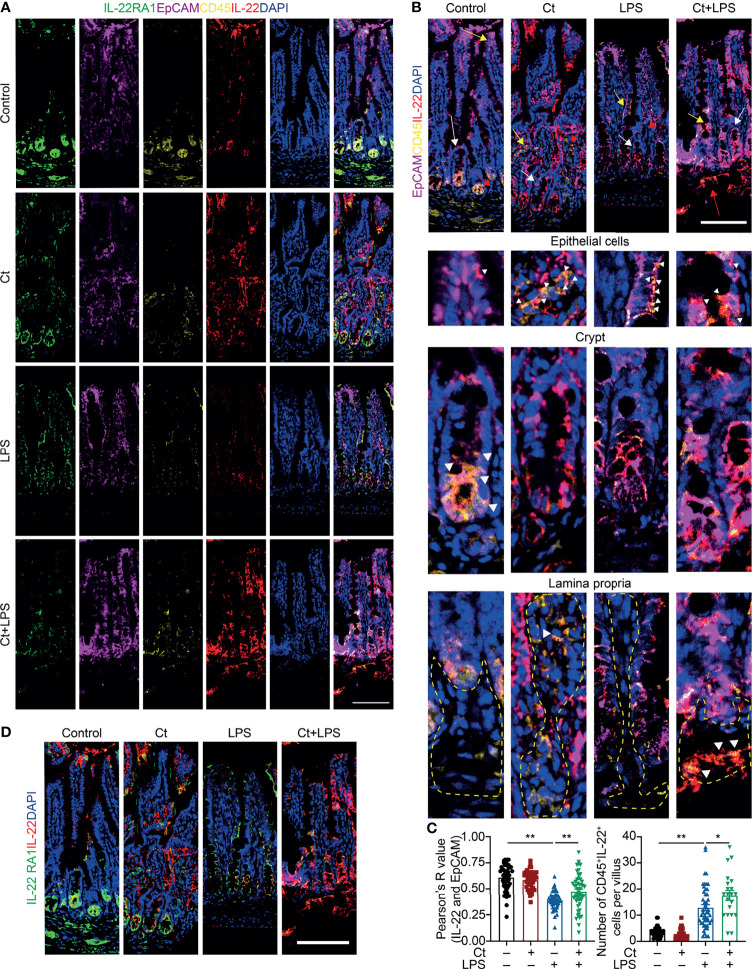
Ct triggers immune cells to enhance the production of IL-22 in the ileal lamina propria. Ileal slices were stained with IL-22 (red), IL-22RA1 (green), EpCAM (violet), CD45 (yellow), and DAPI (blue). **(A)** *In situ* hybridization of IL-22, IL-22RA1, EpCAM, and CD45. **(B)** Colocalization of EpCAM, CD45, and IL-22 in the epithelial position, crypt, and lamina propria, respectively. White triangle: localization, yellow arrow: epithelial cells, white arrow: crypt, red arrow: lamina propria. **(C)** Analysis of IL-22^+^EpCAM^+^ and CD45^+^IL-22^+^ cells. **(D)** Colocalization of IL-22 and IL-22RA1. Scale bar: 100 μm. Data were presented as mean ± SEM. The significant difference was analyzed by two-way unpaired t-tests. *P < 0.05, **P < 0.01.

**Figure 7:**
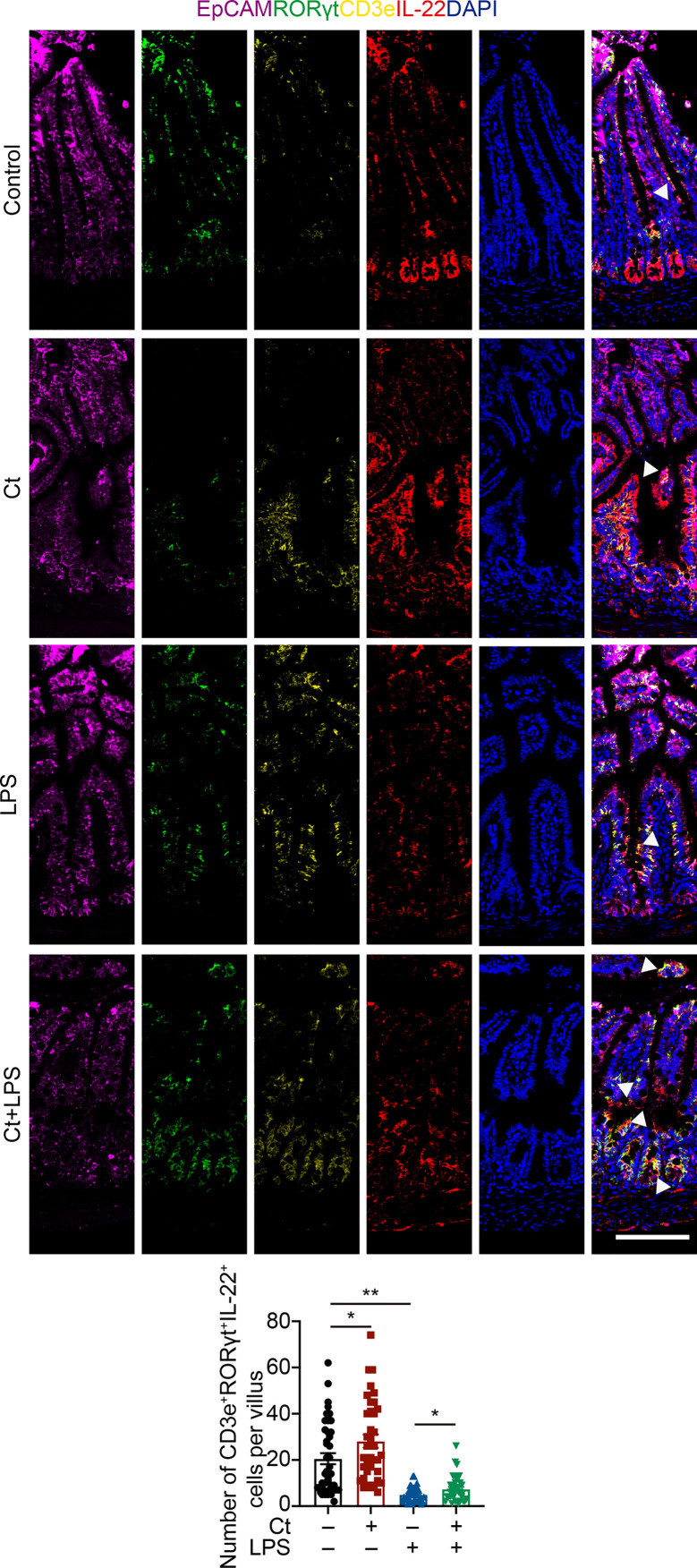
Ct stimulates intestinal Th17 cells to enhance the production of IL-22 in the ileum. Ileal slices were stained with EpCAM (violet), RORγt (green), CD3e (yellow), IL-22 (red), and DAPI (blue). The samples were from the continuous slices in Fig. 6. White triangle: localization. Scale bar: 100 μm. Data were presented as mean ± SEM. The significant difference was analyzed by two-way unpaired t-tests. *P < 0.05, **P < 0.01.

To better understand the origin and position of IL-22 production in the intestine, the ileal slices were stained with EpCAM, CD45, IL-22, and IL-22RA1 (**Figure 6A**). We first analyzed the colocalization of EpCAM, CD45, and IL-22 in the ileum (**Figures 6B, C**). IL-22^+^EpCAM^+^CD45^+^ cells were observed in the intraepithelial lymphocyte and crypt, especially the crypt where IL-22 is highly expressed to promote the proliferation of intestinal stem cells (29, 49). According to the colocalization analysis of EpCAM and IL-22 using Pearson’s R value. In response to LPS, Ct enhanced the colocalization of EpCAM and IL-22, suggesting that the IL-22 colocalized with EpCAM might be from the intraepithelial lymphocytes. Moreover, IL-22^+^CD45^+^ cells were observed in the lamina propria after Ct treatment and Ct enhanced the whole number of CD45^+^IL-22^+^ cells in response to LPS. Ligation of the IL-22-IL-22RA1-IL-10R2 complex is an essential step to mediate the biological effects of IL-22, thereby activating the downstream signaling pathways like the JAK-STAT pathway in epithelial cells and inducing renewal of epithelial cells (38, 50, 51). In the above process, the IL-22RA1 determines the cellular sensitivity to IL-22 and is mainly expressed in outer body barriers of respiratory like lungs, the gastrointestinal like stomach and intestine, the liver, the kidney, but not expressed in the bone marrow, spleen, or thymus containing high proportions of immune cells (52, 53). Herein, we observed that Ct induced higher colocalization of IL-22 and IL-22RA1 in the epithelial cells (**Figure 6D**). Altogether, Ct stimulated the intestinal immune cells to produce IL-22 and triggered the combination of IL-22 with IL-22RA1. The ileal slices were then stained with RORγt, EpCAM, CD3e, and IL-22 to determine whether IL-22 was produced by Th17 cells (**Figure 7**). In response to LPS, Ct enhanced the number of RORγt^+^CD3e^+^IL-22^+^ cells. These results further suggested that Ct enhanced the level of IL-22 induced by Th17 cells in the lamina propria.

### The Role of Ct in Regulating Intestinal Health Was Independent of SCFAs

**Figure 8:**
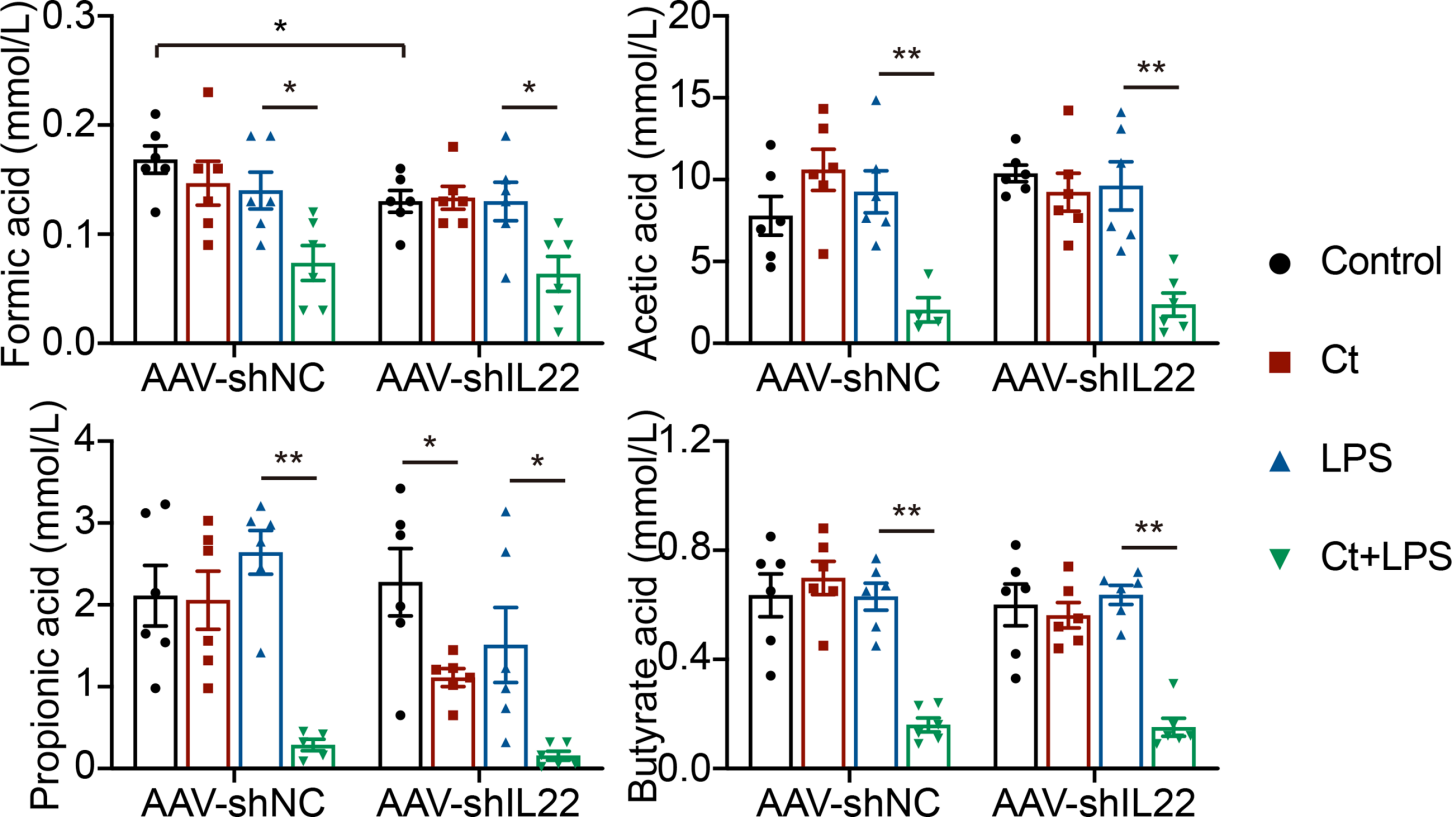
Ct decreases the ileal levels of SCFAs in response to LPS. Data were presented as mean ± SEM (n=6). The significant difference was analyzed by two-way unpaired t-tests. *P < 0.05, **P < 0.01.

Most probiotics regulate intestinal health *via* their metabolites like short-chain fatty acids (SCFAs) in the gut. Bacterial metabolites including short-chain acids (SCFAs), bile acids (BAs), and tryptophan metabolites, bacterial components, and bacteria themselves have been shown to play vital roles in maintaining epithelial integrity and modulating immune responses (54–56). Butyrate acid-producing *Clostridia* like *Clostridium butyrium* has been well studied in regulating intestinal health and inhibiting intestinal tumor development in both patients with IBD and animals with the mechanism of inhibiting histone deacetylase (HDAC) *via* SCFAs (23, 57). Herein, we found that Ct dramatically decreased the levels of SCFAs in the intestine in response to LPS and the decreasing of SCFAs by Ct was independent on IL-22 (**Figure 8**), indicating that Ct protected against intestinal injury in a butyrate acid-independent manner.

## Discussion

Probiotics have been widely used in both humans and production animals. However, some inevitable problems must be considered for probiotics, mainly including the safety, the efficiency in treating intestinal diseases, the colonization in the gut, the viability after passing through the gastrointestinal tract, and the effects on commensal microbiota (58). As for *C. tyrobutyricum*, our previous study has shown that 10^8^ CFU/mL *C. tyrobutyricum* could effectively alleviate LPS-induced inflammation in both the ileum and colon (13, 14). Moreover, the viability of *C. tyrobutyricum* reaches 90.03% after treatment in the simulated gastric fluid *in vitro* (pH 1.2) for 2 h and its viability reaches 62.12% after continuous treatment in the simulated intestinal fluid (pH 7.4) for 4 h (59), suggesting that *C. tyrobutyricum* has considerable high gastrointestinal tolerance. Colonization is an essential characteristic of probiotics. Two decades-long debate views on colonization are existed, namely the colonization of the mucosa during supplementation and the post-supplementation persistence in the gut, which are limited by the abundance of probiotics in the assessment methods directly reflecting their colonization in the gut (58). Herein, we noticed that *C. tyrobutyricum* had the colonization capacity in the small intestine, while the accurate quantitative of viability was not fully revealed because of its weak colonization in the gut. Considering that *C. tyrobutyricum* effectively alleviated intestinal inflammation and prevented LPS-induced diarrhea, how to enhance the colonization or adhesion of *C. tyrobutyricum* on the gut mucosa is an imperative problem in the IBD therapy and the application in preventing diarrhea. A recent study revealed that the bacteria-derived biofilm contributes to the defense of bacteria under extreme conditions, such as physical forces and environmental attacks. The biofilm-coated probiotics present improved gastrointestinal tract tolerance and mucosal adhesion in animals and exhibit higher oral bioavailability and colonization than uncoated bacteria in the intestine (60), providing a feasible method to enhance the colonization or adhesion of *C. tyrobutyricum in vivo*.

According to the transcriptomic analysis, compared with the Control, *C. tyrobutyricum* treatment alone decreased the expression of Cav1, Tacr2, and Adrb3, which were enriched in the process of neuro regulation and calcium signaling pathway according to the KEGG analysis. Moreover, we noticed that mice presented slowly in action during the gavage of *C. tyrobutyricum*. Whether *C. tyrobutyricum* alleviating intestinal dysfunction *via* the brain-gut axis or intestinal nervous regulation needs further study. Considering that LPS induced the metabolic disorder and *C. tyrobutyricum* reversed the metabolic processes like fatty acids synthesis and degradation in response to LPS, whether *C. tyrobutyricum* orchestrating the immune response and metabolism needs to be revealed in the future study.

IL-22, discovered by Gurney’s group and Renauld’s group in 2000 (61, 62), is highly expressed in some gastrointestinal diseases like IBD (27, 63–65). However, IL-22 repairs tissue damage and prevents pathogens *via* promoting the regeneration of epithelial cells and the production of antimicrobial molecules when it is under proper control (38, 45, 46). The fuzzy boundary of IL-22 between inhibitor and inducer for colitis may present a dose-/time-dependent manner in the intestine. In this study, we found that *C. tyrobutyricum* alleviated intestinal inflammation depending on IL-22 produced by Th17 cells. Strangely, LPS stimulation decreased the mRNA expression of IL-6 and proportions of immune cells like macrophages and DCs in the ileum after knocking down IL-22. Some similar results were also observed in the colon (13). Unlike the earlier study in which IL-22 level in the intestine is enhanced in response to LPS (64), herein, the expression of IL-22 in the ileum was decreased in response to LPS. Thus, whether LPS-induced intestinal inflammation depending on IL-22 needs further investigation. More importantly, to fully understand the role of *C. tyrobutyricum* in the intestine, chemical methods like DSS should be considered to induce IBD in our future research.

IECs receive signals from nutrients, commensal microbes, or pathogens, orchestrating the communication between the commensal microorganisms and immune cells under physiological and inflammatory conditions. These signals are classified into three types, namely the microbiota, the microbial components, and the microbial metabolites (2). For most studies, probiotics regulate intestinal health *via* their metabolites like SCFAs, bile acids, and tryptophan metabolites, which have been revealed to maintain the interactions among microbiota, epithelial cells, and immune cells in the gut. The SCFAs activate the G protein-coupled receptors (GPRs) expressed in the epithelial cells to inhibit inflammation *via* producing cytokines like IL-18 (66), concerting the communication between microbiota and IECs. The SCFAs can also regulate the interactions between microbiota and immune cells with the mechanism of stimulating the IL-10 producing Foxp3^+^ Tregs *via* inhibiting HDAC, thereby alleviating colitis (56, 66). As one of *Clostridium* strains producing high levels of butyrate acid *in vitro*, *C. tyrobutyricum* was unable to enhance the levels of SCFAs in the ileum in response to LPS. In this study, we suggested that *C. tyrobutyricum* might play a protective role in the intestine *via* stimulating the metabolites except for SCFAs. Further investigation in whether *C. tyrobutyricum* could trigger the production of metabolites contributing to the stimulation of Th17 cells to produce IL-22 in the gut based on metabolome should be considered.

**Figure 9:**
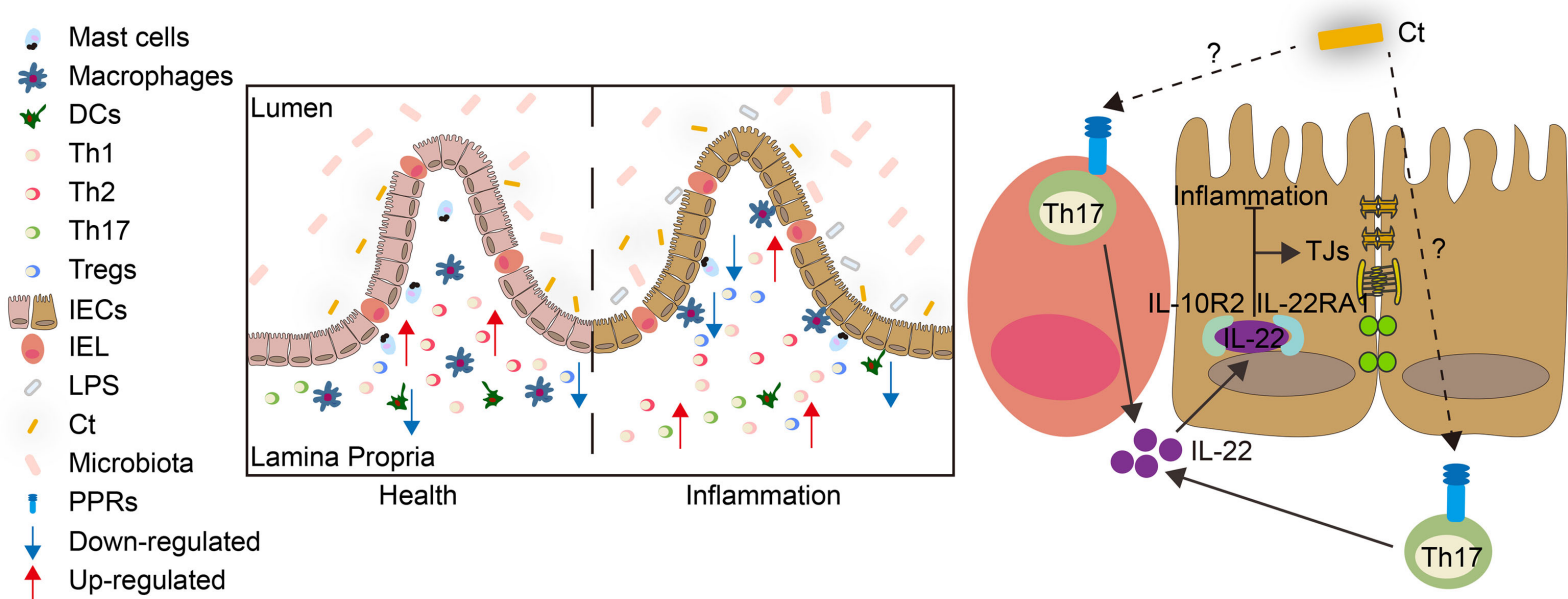
Model for the function and mechanism of Ct in regulating intestinal health. Ct inhibited LPS-induced intestinal inflammation in the ileum. The protective role of Ct in the ileum presented an IL-22-dependent manner *in vivo*. In response to LPS, Ct enhanced the proportions of Th17 cells to produce IL-22, which combined with IL-22RA1 expressed in the epithelial cells, thereby maintaining the barrier function and alleviating inflammation in the ileum.

The present study revealed that *C. tyrobutyricum* alleviated LPS-induced intestinal inflammation and prevented diarrhea in mice with the mechanism of triggering Th17 cells in the lamina propria to induce IL-22 production, which combined with IL-22RA1 expressed in the epithelial cells (**Figure 9**). The results indicated that *C. tyrobutyricum* could be a potential probiotic in regulating intestinal health and provided a scientific basis for its application in preventing diarrhea and inflammation in the ileum.

## Data Availability Statement

The original contributions presented in the study are publicly available. This data can be found here: https://www.ncbi.nlm.nih.gov/sra/?term=PRJNA756726.

## Ethics Statement

The animal study was reviewed and approved by The Institutional Animal Care and Use Committee of Zhejiang University (Approval numbers: ZJU20200005, ZJU20200040).

## Author Contributions

MW took charge of the project administration. ZX designed the whole experiment. ZX and LL performed all *in vivo* and *in vitro* experiments. ZX and LL analyzed and checked the data. ZX wrote the original manuscript. LL revised the manuscript. XP and WS performed the bacterial colonization experiment and cultured the bacteria. YJ performed flow cytometry experiments. ZX, LL, XP, and WS collected the tissue samples. S-TY revised and copy-edited the paper. All authors contributed to the article and approved the submitted version.

## Funding

This study was jointly funded by the National Key Research & Development Program of China (No. 2018YFE0112700) and Key Research & Development Program of Zhejiang Province (No. 2019C02005).

## Conflict of Interest

The authors declare that the research was conducted in the absence of any commercial or financial relationships that could be construed as a potential conflict of interest.

## Publisher’s Note

All claims expressed in this article are solely those of the authors and do not necessarily represent those of their affiliated organizations, or those of the publisher, the editors and the reviewers. Any product that may be evaluated in this article, or claim that may be made by its manufacturer, is not guaranteed or endorsed by the publisher.
