# Supplementary material for: A Potential Probiotic for Diarrhea: Clostridium tyrobutyricum Protects Against LPS-Induced Epithelial Dysfunction via IL-22 Produced By Th17 Cells in the Ileum
Source: Front Immunol. 2021 Nov 30;12:758227. doi: 10.3389/fimmu.2021.758227 (PMC8670534; doi:10.3389/fimmu.2021.758227)
Supplement: Supplementary file 2 [file DataSheet_2.pdf]

## *Supplementary Figures*

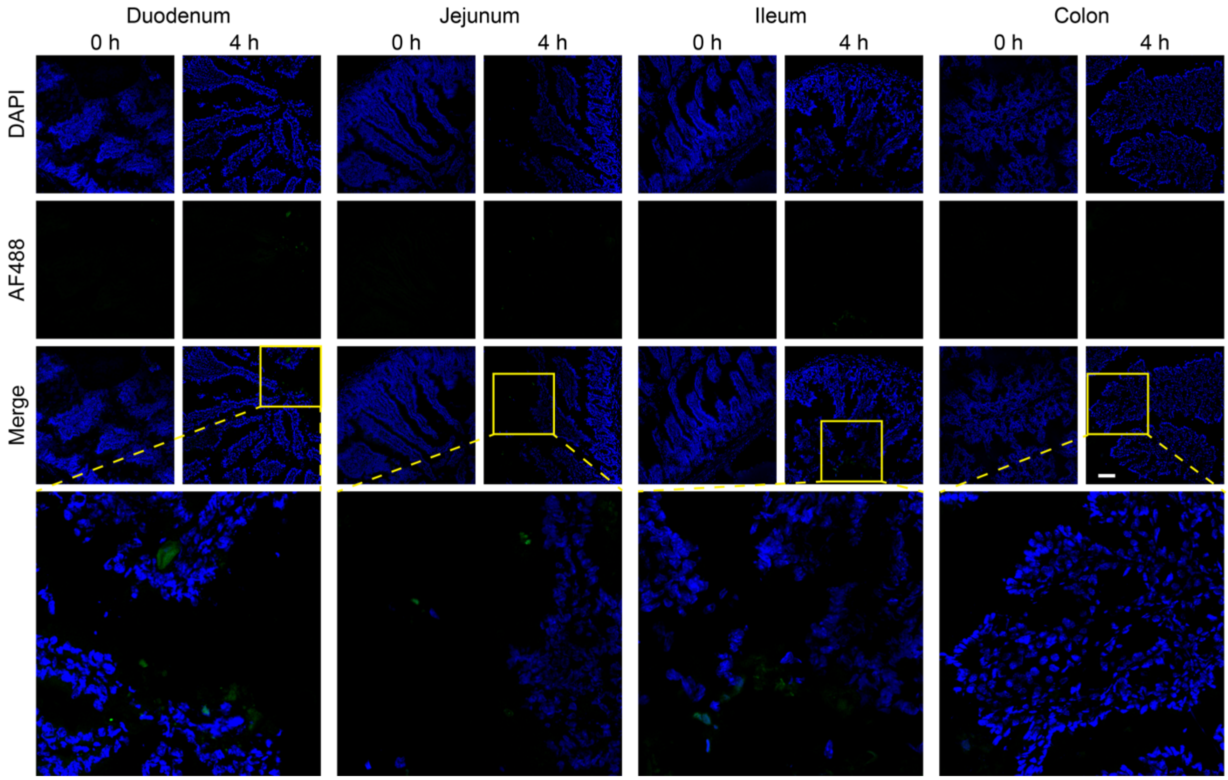


**Supplementary Figure 1. Colonization of Ct in different sections of the small intestine at 4 h.** Ct was labeled with BacLight™ green stain. C57BL/6 mice were treated with $10^8$ CFU/mL Ct by gavage and the intestinal samples were collected at 4 h. Green: Ct, blue: DAPI, scale bar: 50 µm.

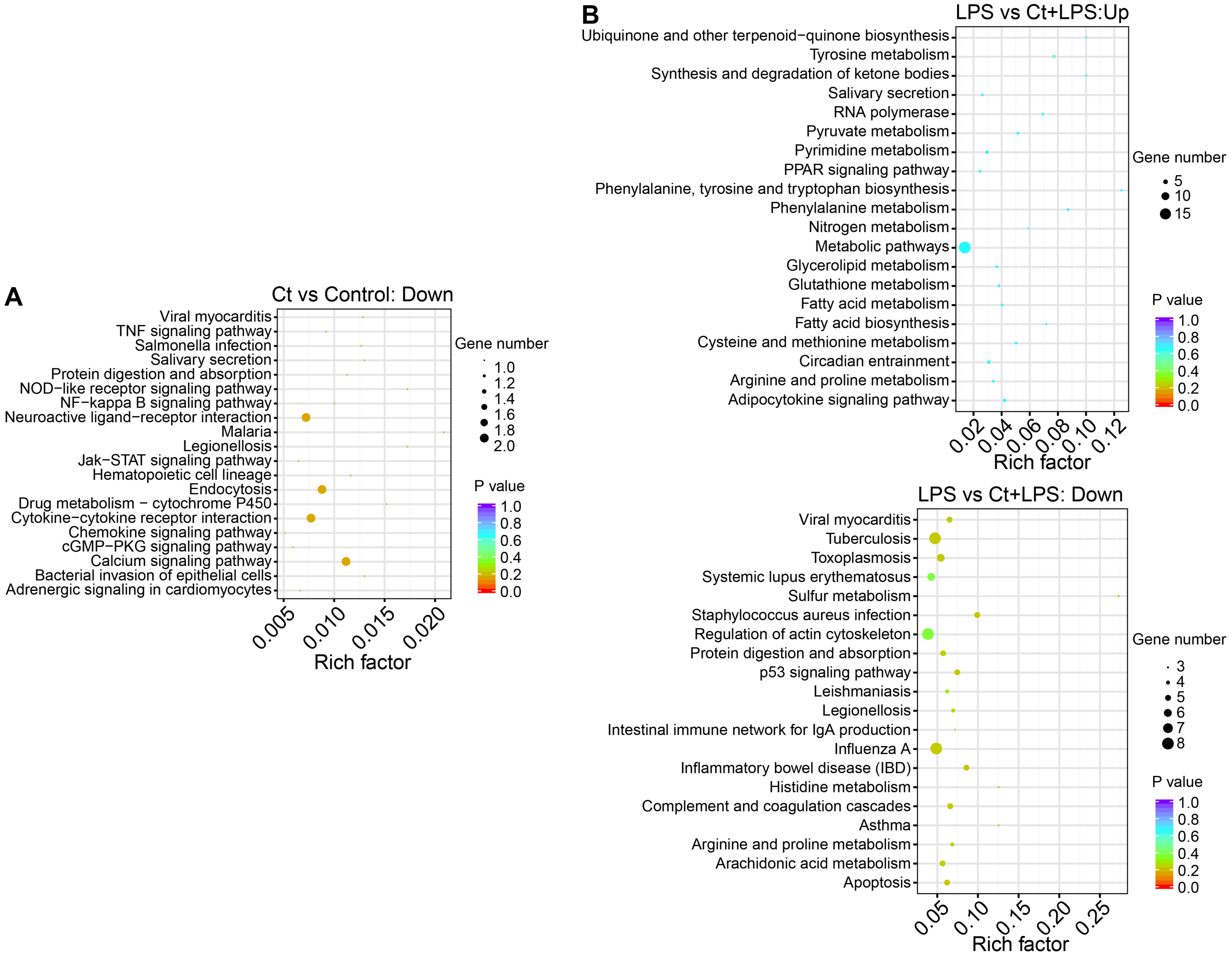


**Supplementary Figure 2. Ct regulates the intestinal nervous system and metabolism in response to LPS. (A)** KEGG pathway enrichment differentially expressed genes between the Control and Ct groups (Ct vs Control, n=6). **(B)** KEGG pathway enrichment differentially expressed genes between the LPS and Ct + LPS groups (LPS vs Ct + LPS, n=6).

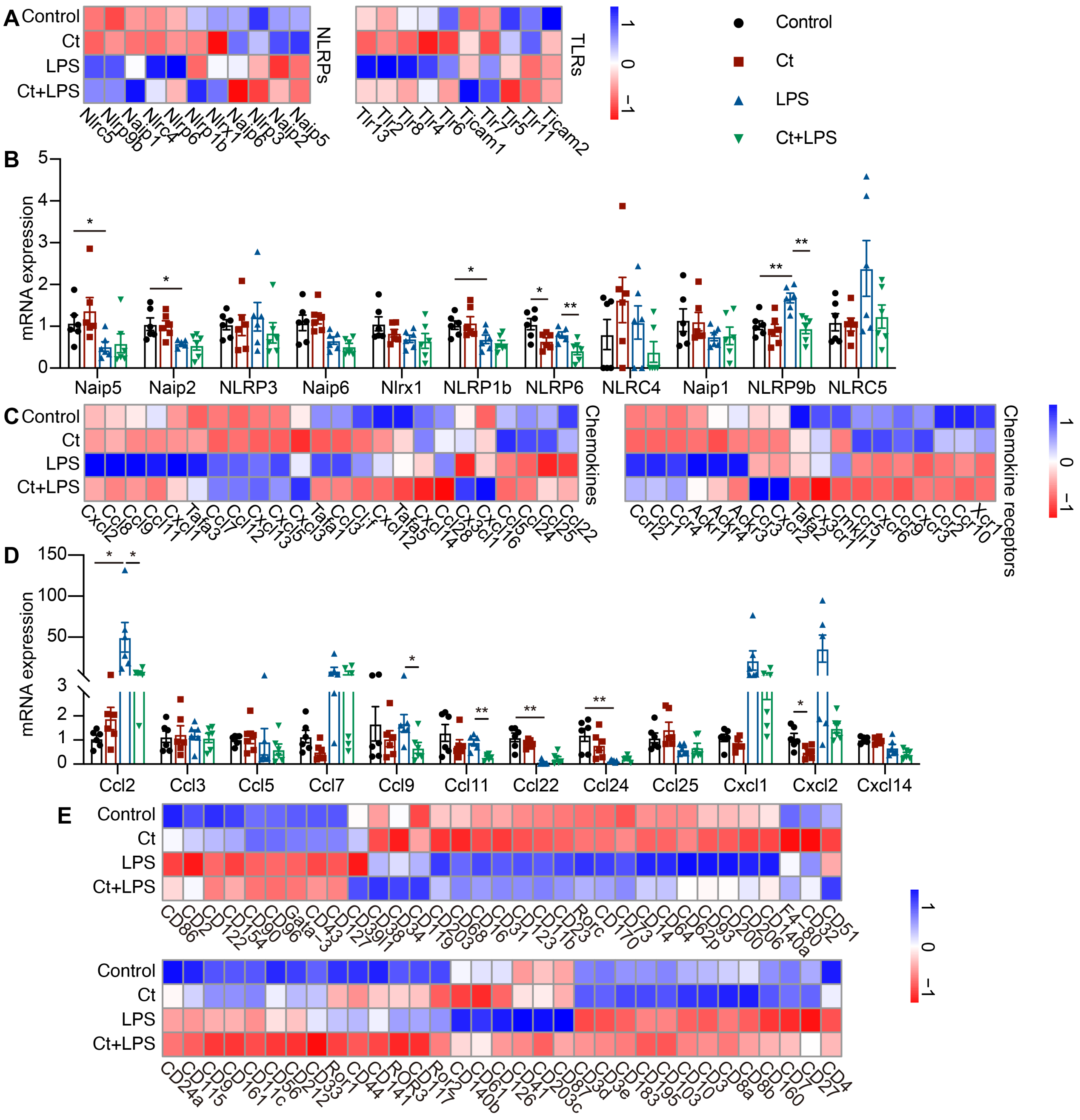

**Supplementary Figure 3. Ct down-regulates the expression of pattern-recognition receptors and regulates intestinal immune cells. (A)** Heat-map of differentially expressed genes enriched in pattern-recognition receptors including TLRs and NLRs. **(B)** RT-qPCR analyses of NLRs in the ileum (n=6). **(C)** Heat-map of differentially expressed genes enriched in chemokines and their receptors. **(D)** RT-qPCR analyses of chemokines in the ileum (n=6). **(E)** Heat-map of differentially expressed genes enriched in the cluster of differentiation. Data were presented as mean ± SEM. The significant difference was analyzed by two-way unpaired t-tests. **P* < 0.05, ***P* < 0.01.

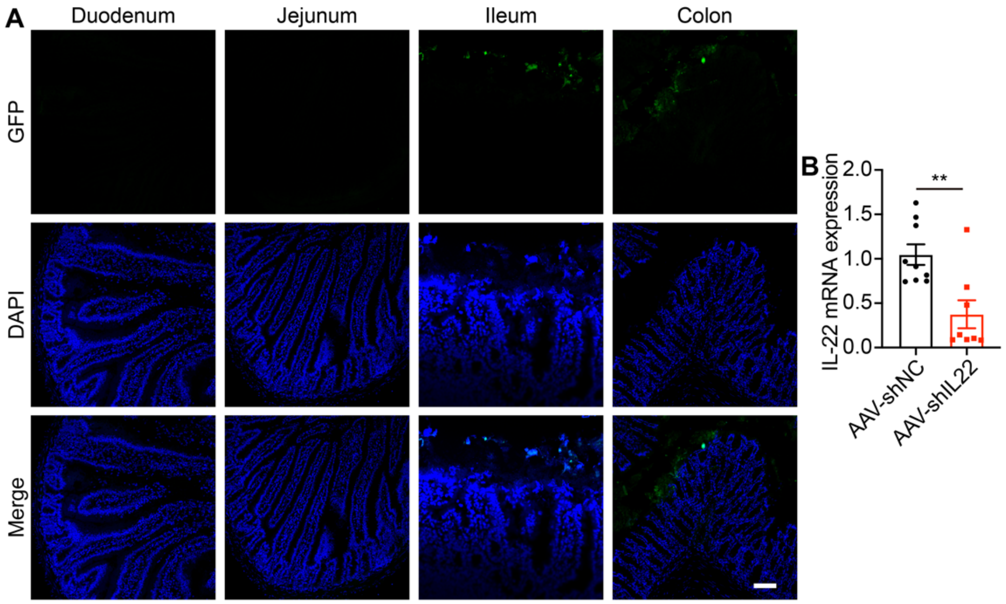


**Supplementary Figure 4. Specificity and efficiency of AAV-shIL22 *in vivo*.** C57BL/6 mice were intraperitoneally injected with AAV-shNC (Vector, n=9) and AAV-shIL22 (n=8) labeled with GFP. The samples were collected after 14 days. **(A)** Fluorescence images of *in vivo* interference in different sections of the intestine. Green: AAV-shIL22-GFP, blue: DAPI, scale bars: 50 μm. **(B)** RT-qPCR analysis of IL-22 in the ileum of the AAV-shNC- and AAV-shIL22-treated mice. Data were presented as mean ± SEM. The significant difference was analyzed by two-way unpaired t-tests. $^*P < 0.05$, $^{**}P < 0.01$.

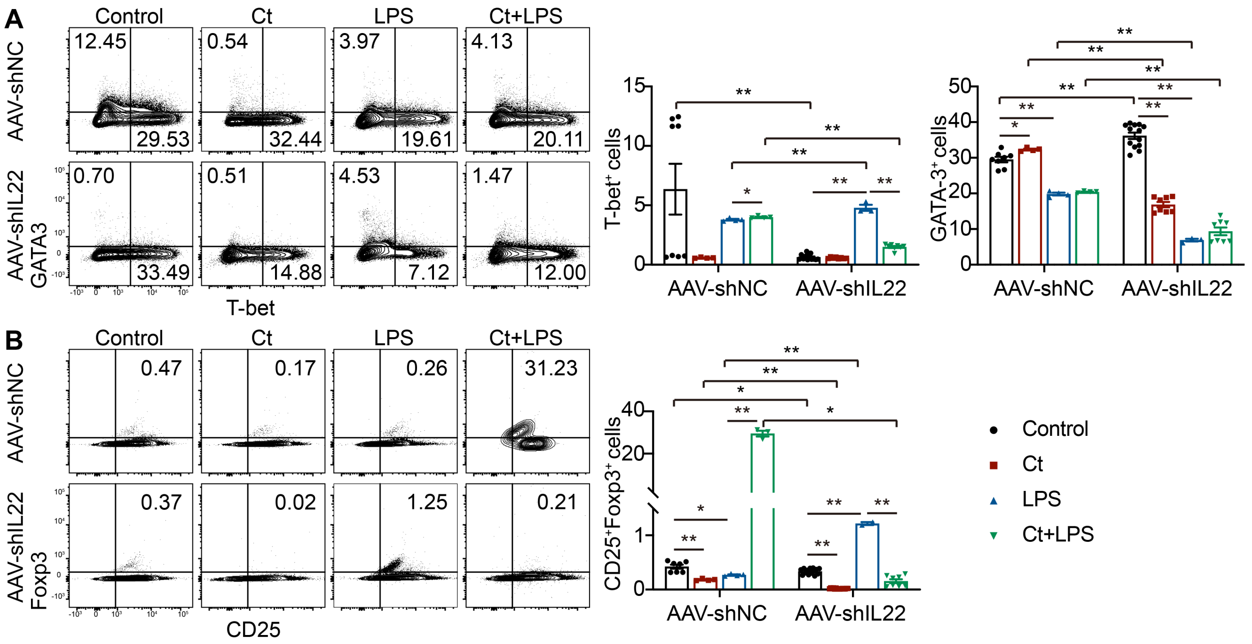


**Supplementary Figure 5. Effects of Ct on the proportions of intestinal immune cells in the ileum. (A)** Proportions of T-bet$^+$ cells (Th1) and GATA$^+$ cells (Th2). **(B)** Proportions of CD25$^+$Foxp3$^+$ cells (Tregs). Each point presented a mouse. Data were presented as mean ± SEM. The significant difference was analyzed by two-way unpaired t-tests. **P* < 0.05, ***P* < 0.01.
